# A Delphi consensus study to identify priorities for improving and measuring medication safety for intensive care patients on transfer to a hospital ward

**DOI:** 10.1093/intqhc/mzac082

**Published:** 2022-10-07

**Authors:** Richard S Bourne, Jennifer K Jennings, Darren M Ashcroft

**Affiliations:** Departments of Pharmacy and Critical Care, Northern General Hospital, Sheffield Teaching Hospitals NHS Foundation Trust, Herries Road, Sheffield S5 7AU, UK; Division of Pharmacy & Optometry, School of Health Sciences, Faculty of Biology, Medicine and Health, The University of Manchester, Oxford Road, Manchester M13 9PL, UK; Departments of Pharmacy and Critical Care, Northern General Hospital, Sheffield Teaching Hospitals NHS Foundation Trust, Herries Road, Sheffield S5 7AU, UK; Division of Pharmacy & Optometry, School of Health Sciences, Faculty of Biology, Medicine and Health, The University of Manchester, Oxford Road, Manchester M13 9PL, UK; National Institute for Health and Care Research (NIHR) Greater Manchester Patient Safety Translational Research Centre (PSTRC), School of Health Sciences, Faculty of Biology, Medicine and Health, University of Manchester, Oxford Road, Manchester M13 9PL, UK; Manchester Academic Health Science Centre, University of Manchester, Oxford Road, Manchester M13 9PL, UK

**Keywords:** Delphi consensus, intensive care, medication usage, medication errors, patient safety

## Abstract

**Background:**

Intensive care patients surviving to transfer to a lower-acuity hospital ward experience ongoing challenges to their recovery and lack a well-defined and developed care pathway. The transfer process to a hospital ward exposes intensive care patients to high rates of medication errors, which increase their risk of adverse drug events.

**Objective:**

The aims of this study were to identify priorities for medication-related intervention components and outcome measures for improving medication safety for intensive care patients transferring to a hospital ward.

**Methods:**

Three panels involving 129 participants covering (i) intensive care, (ii) hospital ward health-care professionals and (iii) public representatives completed an electronic Delphi survey conducted over three phases. The Delphi process comprised three sections (medication-related intervention components, medication outcomes and patient outcomes). Items were graded in their level of importance, with predefined important criteria. Item agreement required consensus across all three panels. Intervention barriers and facilitators identified in participant comments were categorized according to a socio-technical systems approach to the patient journey and patient safety (Systems Engineering Initiative for Patient Safety 3.0 model).

**Results:**

Of the 129 (84.5%) participants, 109 completed all three Delphi phases. Consensus was achieved for 48 intervention components, 13 medication outcome measures and 11 patient outcome measures. Phase 1 provided 158 comments comprising >200 individual barriers and facilitators to intervention delivery. Frequently cited facilitators included clearly specified roles and responsibilities (10.7% (organizational conditions)), patient and family as agents (8.8% (care team)), medicines-related information easily accessible (7.8% (tools and technologies)) and clear medication plan and communication (7.3% (tasks)).

**Conclusions:**

Our findings provide identification of priorities for medication-related intervention components to improve medication safety for intensive care patients transferring to a hospital ward. Prioritization is complemented by the identification and socio-technical categorization of barriers and facilitators to intervention delivery. The identified important medication and patient outcomes to measure will inform the design of a future patient medication safety intervention study.

## Introduction

Intensive care units (ICUs) provide care for critically ill patients in a complex and dynamic clinical environment. Multiprofessional care teams, supported by extensive technological resources, continuously review and adapt patient treatments according to the individual’s illness progression. Medication is similarly regularly reviewed, optimized and adjusted in response to patient requirements. As a result, many changes are made to a patient’s medication during a critical illness episode [1, 2].

ICU patients surviving to transfer to a lower-acuity hospital ward are presented with ongoing challenges to their recovery and lack a well-defined and developed care pathway [3]. Patients frequently encounter adverse events soon after their transfer to a hospital ward, including those related to medicines [4]. Medication errors on patient transfer are common, usually involving the continuation of inappropriate acute medication that is no longer indicated and/or failure to restart important long-term medication [5, 6].

The World Health Organization (WHO) makes several broad recommendations to improve medication safety on transitions in patient care [7]. These include patient and family engagement, medicines reconciliation, improved information communication and availability, medication review, multiprofessional staff collaboration and technological support [7]. In ICU practice, a standardized handover procedure, including an explanation of medication changes and treatment plans, is recommended to improve patient safety on transfer from ICU [8]. In a recent systematic review, we identified the medication-related interventions evaluated to improve medication safety for patients transferring from intensive care settings [9]. The medication safety interventions identified were education of staff, medication review, guidelines, electronic transfer/handover tool or letter and medicines reconciliation [9]. There was a paucity of theory-informed interventions that measured delivery processes or patient outcomes [9]. These are important deficiencies when a complex intervention package is likely to be required to impact on medication safety, acknowledging the complexity of the ICU patient to ward transfer [7, 10]. Routine delivery of such complex interventions in hospital practice requires meaningful and early engagement with appropriate health-care professional stakeholder groups, patients, families or carers to ensure that the intervention is acceptable and implementable at scale across differing system contexts [11, 12]. The development and identification of a complex intervention is needed to improve its validity and reduce the potential for research waste [13]. It is also necessary to ensure that the appropriate medication and patient outcomes are identified for the evaluation of medication-related interventions [7, 11, 13].

The aims of this study were to identify priorities for medication-related intervention components and outcome measures for improving medication safety for ICU patients transferring to a hospital ward.

## Methods

### Candidate items

The Delphi was comprised three sections: (A) medication-related intervention components, (B) medication outcomes and (C) patient (health-care and patient-related) outcomes. Each section was framed to provide an explanation and context for the participants and sub-divided into common themes to assist with participant review and grading. Candidate intervention components (i.e. the individual constituent parts of an intervention) and outcome measures were taken from systematic and scoping reviews identified from the literature and WHO Medication Safety in Transitions of Care technical report [7, 9, 14–17]. Items were described and included a detailed explanation all panel members were likely to understand. The Delphi items were then piloted via members of the United Kingdom Clinical Pharmacy Association (UKCPA) Critical Care Expert Practice Development Group for final content and clarity. Overall, there were 103 items across all three sections in Phase 1 (A = 59, B = 18 and C = 26).

### Participants

Three panels were formed to represent (i) ICU health-care professionals, (ii) hospital ward health-care professionals and (iii) public representatives, providing a broad overview of medication review and prescribing perspectives across the interface in ICU and hospital ward level patient care. For Panels (i) and (ii), health-care professional researchers (identified from authorship of publications exploring ICU patient transitions, including a medication safety element) [6, 9, 14–17], were invited to participate and to share the invitation with appropriate professional colleagues. Next, health-care professionals from developed countries, with appropriate expertise (in medication review of ICU patients transitioning from ICU), were identified via ICU, pharmacy and medical professional organization adverts targeted at the relevant sections of the Canadian Society of Hospital Pharmacists, Faculty of Intensive Care Medicine, Intensive Care Society, National Outreach Forum, Royal College of Physicians, Royal Pharmaceutical Society, Society of Critical Care Medicine, Society of Hospital Pharmacists of Australia, UK Critical Care Research Group and UKCPA. Finally, advertisements for public participants were sent to key UK ICU and emergency care patient and family groups (Intensive Care Society Patients and Relatives Professional Advisory Group, ICU Steps and Critical Care Support Network), as well as selected local and regional groups. All participants provided written informed consent. Only the public panel participants received any remuneration for their participation in the completed phases.

### Delphi process and phases

The Delphi process was conducted electronically, providing participants with remote and convenient access to a bespoke Delphi platform (National Perinatal Epidemiology Unit, University of Oxford). For each item, participants were asked to score the ‘importance’ from 1 to 9 in increasing level of importance, where 1, 2 and 3 were ‘not that important’; 4, 5 and 6 were ‘important’ and 7, 8 and 9 were ‘really important’. Participants were encouraged to score an item ‘don’t know’ if they felt an item was beyond their expertise. This item importance grading approach was based on the recommendations from the Core Outcome Measures in Effectiveness Trials (COMET) initiative [18]. Panel consensus on ‘important’ criteria was met when ≥70% of participants scored 7–9 and <15% of participants scored the item 1–3; ‘Not important’ criteria was met when ≥70% of panel participants had scored 1–3 and <15% of participants had scored the item 7–9. An item was agreed as ‘important’ if it met the relevant ‘important’ criteria for all three panels (ICU, Ward and Public).

In Phase 1, participants could also comment on the items and make recommendations for any changes or additions. In Phase 2, panel members saw graphical and numerical representations of their own grading and the panel median, providing the opportunity for them to review their grades. In Phase 3, graphical and numerical displays of all three-panel medians were shown, providing a final opportunity for participants to review their item gradings. All free-text comments were reviewed by two investigators (RSB and JKJ) for action. Participants were sent up to four reminders to complete each phase. Those participants who had not fully completed the phase within 3 weeks of launch were removed from the subsequent phase.

### Data analysis

For each phase, we calculated for each item (excluding the ‘don’t know’):

(i) each panel’s median (IQR) score; (ii) the percentage of each panel scored an item 1–3 and 7–9; (iii) the number of panels where ≥70% of participants scored 7–9 and <15% of participants scored the item 1–3 and (4) the number of panels where ≥70% of participants scored 1–3 and <15% of participants scored the item 7–9.

The barriers and facilitators to intervention components, identified by participants in their free-text comments, were categorized in terms of a socio-technical systems approach to the patient journey and patient safety [19]. Categories represented the work system (care team, organizational conditions, tools and technologies, tasks and physical environment) from the Systems Engineering Initiative for Patient Safety 3.0 model [19].

## Results

One-hundred and twenty-nine participants consented to participate in the Delphi process. The ICU panel included the most participants (87, 67.4%), and clinical pharmacists were the largest group represented (83, 64.3%) (Table 1). More than 80% of those enrolled were based in the UK (Table 1). Of those that consented, 123 (95.3%) participants completed Phase 1, and 113 (87.6%) participants completed Phase 2. The low attrition rate continued into Phase 3, with 109 participants (84.5%) completing all three phases.

**Table 1:** Participants enrolled in the Delphi process and geographical representation

	ICU panel	Hospital ward panel	Public panel	Country/region	Number (%)
Clinical pharmacist	70	13	N/A	UK	106 (82.2)
Medical staff	11	3	N/A	USA	8 (6.2)
Advanced practitioner	4	5	N/A	Australia	6 (4.7)
Nursing staff	2	-	N/A	Canada	5 (3.9)
Other	-	1	N/A	Europe	2 (1.6)
Public	N/A	N/A	20	Middle East	2 (1.6)
Total	87	22	20	Total	129 (100)

### Phase 1

Two-hundred and fifty-seven comments were received from 120 participants in Phase 1. Of these, 158 (61.5%) were related to the medication intervention components. The remaining comments reaffirmed or explained the basis for the participant’s item grading and provided examples of local practice or experience or provided further context on local resources, systems and barriers or facilitators to implementation. Two-hundred and five individual barriers or facilitators were identified from the comments made by participants in Phase 1 (Table S1 of Supplementary File 1). The most frequently cited facilitators were clearly specified staff roles and responsibilities (10.7% (organizational conditions)), patient and family as agents (8.8% (care team)), medicines-related information easily accessible (7.8% (tools and technologies)) and clear medication plan and communication (7.3% (tasks)).

For Phase 2, one further item was added, and the explanations for four more items were clarified in response to comments received. Clarification involved emphasizing the potential link between medication-related adverse events and complications such as readmissions. The additional item was within the medication-related intervention components section (A) (‘All appropriate ICU clinical staff aware of which clinical specialty the patient’s care is being transferred to’).

### Phase 2

In Phase 2, there was agreement across all three panels on the importance of 62 of 103 items (60.2%). None of the items met the ‘not important’ criteria. The remaining 41 items were kept for consideration by participants in Phase 3.

### Phase 3

**Figure 1:**
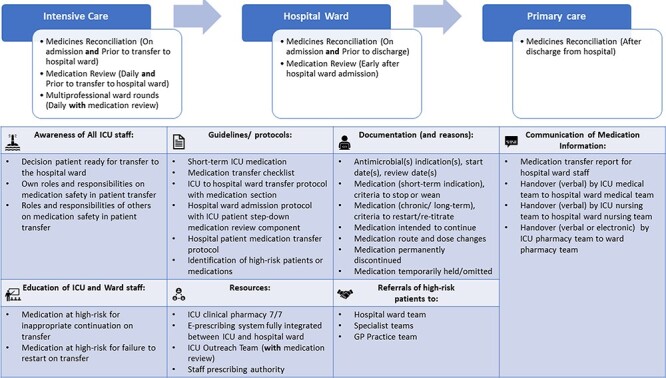
Graphical representation of all agreed important intervention components and timing of medication review and reconciliation in the patient journey.

After Phase 3, 10 further items met the consensus criteria for importance, so overall 72 (69.9%) items were agreed by all panels as important. Forty-eight important intervention components were agreed, with 13 medication outcome measures and 11 patient outcome measures deemed important. The medication-related intervention component item added after Phase 1 did not achieve consensus across all panels. The final agreed important items for each section are shown in Table 2. A graphical representation of all important intervention components is shown in Figure 1. Ten other items were deemed important by two of the three panels, with either the ICU or ward panel not reaching consensus on importance on five occasions each (Table S2 of Supplementary File 1). Results from all phases are presented in Supplementary File 2.

**Table 2:** All items meeting important criteria across all three panels

Section 1	Medication-related components	Explanation
A	ICU clinical team organization and education of staff	
1	All appropriate ICU clinical staff are aware of the decision that the patient is deemed ready for transfer from the ICU to a hospital ward	Once the clinical decision is made that a patient is ready for transfer from the ICU to a hospital ward, all appropriate ICU health-care professionals are aware so that planning of continuity of care (including medication) can be done
2	All ICU clinical staff are aware of their own specific roles and responsibilities in the safety and continuity of medication in ICU patients on transfer to a hospital ward	All ICU health-care professionals understand their specific roles in medication safety and continuity of ICU patients on transfer to a hospital ward
3	All ICU clinical staff are aware of the specific roles and responsibilities of other health-care professionals in the safety and continuity of medication in ICU patients upon transfer to a hospital ward	All ICU clinical staff are aware of specific roles and responsibilities (including specific task allocation) of the other health-care professionals involved in the safety and continuity of medication for ICU patients on discharge to a hospital ward
4	Multiprofessional ward rounds are undertaken daily on ICU with attendance by all appropriate health-care professions involved in patient medication review	Multiprofessional ward rounds provide the opportunity for all health-care professionals (i.e. ICU medical, nursing, pharmacy, microbiology and physiotherapy staff) attending to actively input into and be fully aware of the ongoing patient care plan including medication
5	Education of ICU staff on medication at high risk for inappropriate continuation on transfer to a hospital ward (e.g. stress ulceration prophylaxis, antipsychotics for delirium, antiarrhythmics and bronchodilators)	ICU staff involved in the ICU patient ward transfer process are educated on common medication errors when short-term medication is inappropriately continued on ICU patient transfer despite a lack of ongoing indication. These commonly include stress ulceration prophylaxis, antipsychotics or sedatives for delirium symptoms, antiarrhythmic medication for short-term heart rhythm problems and bronchodilators for chest wheeze
6	Education of ICU staff on medication at high risk for failure to restart on transfer to a hospital ward (e.g. antiplatelets, anticoagulants, mental health medication and cardiovascular medication)	ICU staff involved in the ICU patient ward transfer process are educated on common medication errors when important chronic medication is either not restarted when indicated or a review planned for ward staff to recommence once the patient is clinically appropriate to do so. These medicines commonly include antiplatelets, anticoagulants (‘blood thinners’), mental health medication and cardiovascular (‘heart’) medications
7	Education of hospital ward staff on medication at high risk for inappropriate continuation on transfer to a hospital ward (e.g. stress ulceration prophylaxis, antipsychotics for delirium and bronchodilators)	Hospital ward staff involved in the ICU patient ward transfer process are educated on common medication errors when short-term medication is inappropriately continued on ICU patient transfer despite a lack of ongoing indication. These commonly include stress ulceration prophylaxis, antipsychotics or sedatives for delirium symptoms, ant-arrhythmic medication for short-term heart rhythm problems and bronchodilators for chest wheeze
8	Education of hospital ward staff on medication at high risk for failure to restart on transfer to a hospital ward (e.g. antiplatelets, anticoagulants, mental health medication and cardiovascular medication)	Hospital ward staff involved in the ICU patient ward transfer process are educated on common medication errors when important chronic medication is either not restarted when indicated or a review planned for ward staff to recommence once the patient is clinically appropriate to do so. These medicines commonly include antiplatelets, anticoagulants (‘blood thinners’), mental health medication and cardiovascular (‘heart’) medications
B	Guidelines or protocols	
9	Guidelines (ICU/ hospital) on short-term ICU medication, including indication and when to stop or wean off	Paper or electronic guidelines available and effectively implemented so ICU and/or ward health-care professionals are supported in the starting and stopping short-term medication commonly commenced in ICU, e.g. stress ulceration prophylaxis and antipsychotics in delirium
10	Medication checklist on ICU patient transfer to a hospital ward (is recommended)	A medication checklist is recommended for use by ICU staff to complete with prompts provided for the key medication components
11	Medication checklist on ICU patient transfer to a hospital ward (mandatory)	A medication checklist is mandatory for use by ICU staff to complete with prompts for the key medication components. ICU staff cannot complete the ICU patient transfer to the hospital ward without completing the checklist
12	ICU to hospital ward transfer protocol with medication section	A local protocol that includes all the medication safety systems and processes required to complete the ICU patient transfer to a hospital ward
13	Hospital ward admission protocol with ICU patient step-down medication review component	A local protocol that includes all the medication safety systems and processes required to admit a patient to a ward from ICU
14	Hospital patient medication transfer protocol	A local protocol that includes all the medication safety systems and processes required to complete the patient discharge from the hospital to the community or other non-hospital destination
15	Guideline on identification of high-risk patients or medications	Guideline to aid clear identification of high-risk patients based on their co-morbidities (pre-existing illnesses) (e.g. past medical history, acuity of illness and duration of ICU stay) or medication (e.g. polypharmacy, medication at high risk of error and medicines needing review or monitoring)
C	Medication-specific tasks or processes	
16	Medication (short-term indication), documentation of criteria to stop or wean	Depending on short-term medication indication, duration of treatment and patient symptoms, criteria to prompt ICU and ward staff to either stop or if weaning/tapering are required before stopping, e.g. antipsychotics for delirium symptoms that have resolved
17	Medication (chronic/long term), documentation of criteria to restart/re-titrate	Depending on chronic/long-term medication indication and patient condition, criteria to prompt ICU and ward staff to either restart or re-titrate medication, e.g. re-titration of Angiotensin-Converting Enzyme (ACE)-inhibitor in a patient with heart failure recovered from an acute kidney injury
18	Medication intended to continue is documented	Documentation of medication intended to be continued long term
19	Medication route changes are documented	Documentation of medication with route changes (short or long term), e.g. intravenous medication while unable to take oral medication
20	Medication dose changes and reasons are documented	Documentation of medication with dose changes and reasons, e.g. b-blocker dose increased as the patient had episodes of fast-AF (atrial fibrillation, irregular heartbeat) now resolved
21	Medication is permanently discontinued and reasons are documented	Documentation of medication intended to be permanently discontinued, e.g. bendroflumethiazide (water tablet), and reason, e.g. severe hyponatraemia (low salt levels in blood), documented
22	Medication is temporarily held/omitted and reasons are documented	Documentation of medication temporarily held/omitted and reasons, e.g. ACEI (heart failure or high blood pressure medication) during acute kidney injury episode (kidney failure)
23	Medicines reconciliation on admission to ICU	Completion and documentation of the best possible medication history for the patient on admission to ICU. Usually completed from multiple sources including the patient or family
24	Medicines reconciliation on ICU prior to transfer to the hospital ward	Completion and documentation of the best possible medication history for the patient on ICU prior to transfer to a hospital ward. Usually completed from multiple sources, including the patient, family or carers, and ICU medication records
25	Medicines reconciliation on the hospital ward after ICU to hospital ward transfer	Completion and documentation of the best possible medication history for the patient on the receiving ward transfer after ICU transfer. Usually completed from multiple sources, including the patient, family or carers, and ICU medication records
26	Medicines reconciliation prior to discharge from the hospital	Completion and documentation of the best possible medication history for the patient prior to discharge from the hospital. Usually completed from multiple sources, including the patient, family or carers, and hospital medication records
27	Medicines reconciliation in the general practice (GP) practice after discharge from the hospital	Completion and documentation of the best possible medication history for the patient in the GP practice (primary care physician/practice) after hospital discharge. Usually completed from multiple sources, including the patient, family or carers, and hospital medication records
28	Medication review—undertaken daily on ICU	A structured evaluation of a patient’s medicines with the aim of optimizing medicines use and improving health outcomes. Involves a critical review of current medication (ideally with the patient or family); ongoing medication requirements and tolerance of medication, with the aim of improving medication outcomes. Undertaken at least once per day on ICU
29	Multiprofessional ICU ward round includes patient medication review—undertaken daily on ICU	ICU health-care professionals meet daily to review and plan each ICU patient’s care, including undertaking a medication review element (a structured evaluation of a patient’s medicines with the aim of optimizing medicines use and improving health outcomes)
30	Medication review—on ICU prior to transfer to a hospital ward	A structured evaluation of a patient’s medicines with the aim of optimizing medicines use and improving health outcomes. Involves a critical review of current medication (ideally with the patient or family); ongoing medication requirements and tolerance of medication, with the aim of improving medication outcomes. Undertaken on ICU in advance of the transfer to a hospital ward
31	Medication review on the hospital ward soon after transfer to a hospital ward	A structured evaluation of a patient’s medicines with the aim of optimizing medicines use and improving health outcomes. Involves a critical review of current medication (ideally with the patient or family); ongoing medication requirements and tolerance of medication, with the aim of improving medication outcomes. Undertaken on the ward, within 48 h of transfer from ICU
D	Resources and infrastructure (additional those routinely expected in a high economy, e.g. UK)	
32	Electronic prescribing systems in ICU and hospital ward are fully integrated	Electronic prescribing and medicines administration systems work seamlessly throughout the hospital (including ICU and hospital ward), so that prescribed or held/omitted medication status is maintained across the care interfaces. Some ICUs and hospital wards have different electronic prescribing systems that may not ‘talk’ to each other
33	ICU clinical pharmacy services are provided 7 days/week	ICU clinical pharmacist available 7 days/week in the clinical area to support medication safety and quality of use
34	ICU outreach team that follow up ICU patients on transfer to a hospital ward include a medication review component	ICU team specializing in patient care on the interfaces of ICU that follow up ICU patients recently transferred to a hospital ward and undertake a patient medication review as part of that follow up
35	Health-care professionals involved in the medication review of ICU patients have appropriate prescribing authority to their role	All medical, advanced nurse practitioners and clinical pharmacists involved with medication review on the interface of ICU patient transfer to a hospital ward have the ability to make appropriate changes to the patient’s medication therapy by prescribing authority (e.g. independent prescribing) in response to the medication review undertaken
E	Referral to other health-care professionals or teams	
36	High-risk patients for medication-related problems (e.g. medication continuity or transfer errors) identified and planned/referred for follow-up by the hospital ward team	High-risk patients for medication-related problems (e.g. medication continuity or transfer errors) identified and planned/referred for follow-up by the hospital ward team
37	High-risk patients for medication-related problems (e.g. medication continuity or transfer errors) identified and planned/referred for follow-up by the GP practice	High-risk patients for medication-related problems are easily identified, e.g., by polypharmacy measure or high-risk medication and prioritized for a medication review by the GP practice (primary care physician/practice)
38	Referral to a specialist team for a medication review (e.g. diabetes, care of the elderly, psychiatry, cardiology and acute pain teams)	For specific medication or clinical scenarios, a referral is made for a specialist health-care team to follow the patient up on the ward to support medication review and continuity of therapy, e.g. patients on insulin the diabetes team, patients with ongoing delirium (care of the elderly or psychiatry teams) and acute pain problems (acute pain team)
F	Information transfer/communication	
39	Antimicrobial(s) indication(s) included	Why any antimicrobial(s) (medicines used to treat an infection) continuing after ICU patient transfer to a hospital ward were started
40	Antimicrobial start dates included	Start date(s) of any antimicrobial(s) (medicines used to treat an infection) continuing after ICU patient transfer to a hospital ward
41	Antimicrobial review dates included	Review date(s) of any antimicrobial(s) (medicines used to treat an infection) continuing after ICU patient transfer to a hospital ward
42	Medication report for the ICU patient on transfer to a hospital ward to hospital ward staff	A medication report/letter (paper or electronic) on ICU patient transfer to a hospital ward that contains the agreed medication information for ward staff use
43	Handover (verbal) of medication therapy information and review requirements by ICU medical team to hospital ward medical team	A verbal (e.g. telephone call) handover from the ICU to the hospital ward medical teams using a structured format (e.g. SBAR—Situation Background Assessment Recommendation) that includes a medication review element when needed
44	Handover (verbal) of medication therapy information, review and administration requirements by ICU nursing team to hospital ward nursing team	A verbal (e.g. telephone call) handover from the ICU to the hospital ward nursing teams using a structured format (e.g. SBAR) that includes a medication review element when needed
45	Handover (verbal or electronic) of medication therapy information and review requirements by ICU pharmacy team to ward pharmacy team	A verbal (e.g. telephone call) or electronic (e.g. electronic message) handover from the ICU to the hospital pharmacy medical teams using a structured format (e.g. SBAR) that includes a medication review element when needed
**Section 2**	**Medication-related outcomes**	**Explanation**
A	Polypharmacy measures	
46	Number of changes in high-risk medicines on ICU transfer to the hospital ward	Includes all changes to high-risk medicines (high-risk medicines are those that have an increased risk of causing significant patient harm when they are used in error), doses and routes on ICU transfer to the hospital ward. Compared to ICU pre-admission medicines reconciliation. To provide an indication of the risk and complexity of medication changes at ICU transfer. High-risk medicines for medication errors, e.g. diuretics or opioids/narcotics
47	Number of changes in medicines on hospital discharge	Includes all changes to medicines, doses and routes on hospital discharge. Compared to ICU pre-admission medicines reconciliation. To provide an indication of the complexity of medication changes at hospital discharge
48	Number of changes in high-risk medicines on hospital discharge	Includes all changes to high-risk medicines (high-risk medicines are those that have an increased risk of causing significant patient harm when they are used in error), doses and routes on hospital discharge. Compared to ICU pre-admission medicines reconciliation. To provide an indication of the risk and complexity of medication changes at hospital discharge. High-risk medicines for medication errors, e.g. diuretics or opioids/narcotics
B	Medication error measures	
49	Number of medication errors on ICU transfer to the hospital ward	Count of any medication errors identified on the interface of the patient transferring from ICU to the hospital ward. Medication errors are defined as ‘a failure in the treatment process that leads to, or has the potential to lead to, harm to the patient’
50	Number of medication errors on hospital discharge	Count of any medication errors identified on hospital discharge. Medication errors are defined as ‘a failure in the treatment process that leads to, or has the potential to lead to, harm to the patient’
51	Clinical severity of medication errors on ICU transfer to the hospital ward	For each medication error on ICU to hospital ward transfer, a clinical impact rating for the error as if it would have potentially affected patient care
52	Clinical severity of medication errors on hospital discharge	For each medication error on hospital discharge, a clinical impact rating for the error as if it had been allowed to affect patient care
53	Number of clinically important ‘moderate’ or higher medication errors on ICU transfer to the hospital ward	Count of any medication errors of ‘moderate’ or higher potential patient harm identified on the interface of the patient transferring from ICU to the ward. Harm Associated with Medication Errors Classification (HAMEC): ‘Moderate’ = Potential for minor, non-life-threatening, temporary harm that would require efforts to assess for a change in a patient’s condition such as monitoring and additional low-level change in a patient’s level of care such as a blood test. Any potential increase in the length of care is likely to be minimal (<1 day)
54	Number of clinically important ‘serious’ or higher severity medication errors on ICU transfer to the hospital ward	Count of any medication errors of ‘serious’ or higher potential patient harm identified on the interface of the patient transferring from ICU to the hospital ward. HAMEC: ‘Serious’ = Potential for major, non-life-threatening, temporary harm or minor permanent harm that would require a high level of care such as the administration of an antidote. An increase in the length of care of ≥1 day is expected
55	Number of clinically important ‘moderate’ or higher severity medication errors on hospital discharge	Count of any medication errors of ‘moderate’ or higher potential patient harm identified on hospital discharge. HAMEC: ‘Moderate’ = Potential for minor, non-life-threatening, temporary harm that would require efforts to assess for a change in a patient’s condition such as monitoring and additional low-level change in a patient’s level of care such as a blood test. Any potential increase in the length of care is likely to be minimal (<1 day)
56	Number of clinically important ‘serious’ or higher severity medication errors on hospital discharge	Count of any medication errors of ‘serious’ or higher potential patient harm identified on hospital discharge. HAMEC: ‘Serious’ = Potential for major, non-life-threatening, temporary harm or minor permanent harm that would require a high level of care such as the administration of an antidote. An increase in the length of care of ≥ 1 day is expected
57	Categorization of medication errors (e.g. by psychological approach)	This categorization system divides errors into mistakes (rule or knowledge-based), slips or lapses. The categorization helps to identify systems to reduce or prevent such errors happening again
C	User satisfaction with information provision	
58	Hospital ward multiprofessional team satisfaction with the transfer of medication information and plan (from ICU to ward)	Members of the hospital ward multiprofessional team (e.g. medical, nursing and pharmacy staff) degree of satisfaction with the medication information and plan received with an ICU patient transfer to a hospital ward
**Section 2**	**Medication-related outcomes**	**Explanation**
59	GP practice staff satisfaction with medication information and plan (from hospital)	Members of the GP practice health-care professional team (e.g. medical, nursing and pharmacy staff) degree of satisfaction with the medication information and plan received from the hospital with an ICU patient discharge
60	Patient and/or family satisfaction with the ICU medication transfer information and plan (from ICU and hospital)	Patient and/or family degree of satisfaction with the medication information and plan received with the ICU patient transfer to a hospital ward and discharge from the hospital
61	Time to first medication review after ICU patient transfer to a hospital ward	Time (h) to first medication review (a structured evaluation of a patient’s medicines with the aim of optimizing medicines use and improving health outcomes. Involves a critical review of current medication (ideally with the patient or family); ongoing medication requirements and tolerance of medication, with the aim of improving medication outcomes) after transfer to a hospital ward
**Section 3**	**Patient-related outcomes**	**Explanation**
A	Adverse drug events	
62	Adverse drug events on the hospital ward after ICU transfer (number documented for patient)	An adverse drug event (an injury resulting from medical intervention related to a drug) occurring in the patient on the hospital ward after ICU transfer. Count of medical records
63	Preventability of the adverse drug event after ICU transfer	Preventable (related to an error) and non-preventable (not predictable)
64	Severity of actual harm associated with the adverse drug events occurring on the ward after ICU transfer	The degree of actual patient harm caused by an adverse drug reaction. Categorized by a validated scale, e.g. HAMEC
65	Adverse drug event attributed to ICU or hospital ward care (number documented for patient)	Attempt to attribute origin of cause of adverse drug event either at ICU or hospital ward level
B	Clinical frailty and quality of life	
66	Clinical frailty scale on ICU admission (older people only, >65 years)	A clinical measure of an older (>65 years) person’s level of vulnerability to poor outcomes. The clinical frailty scale range is from 1—very fit—to 9—terminally ill. Assessed on activities 2 weeks prior to ICU admission. To provide a baseline patient frailty assessment for comparison later
C	Readmissions	
67	Timing of ICU patient transfer to a hospital ward	Actual time and day of week and timing classified as either ‘in hours’ (e.g. Monday–Friday 07:00–21:59) or outside of normal hours (e.g. Monday–Friday 22:00–06:59 or on weekends)
68	Readmission rate to ICU (within 48 h)	Patients readmitted to ICU, possibly due to a medication-related complication, within 48 h of ICU transfer to the hospital ward
69	Readmission rate to ICU (within 7 days)	Patients readmitted to ICU, possibly due to a medication-related complication, within 7 days of ICU transfer to the hospital ward
70	Resuscitation team call on ward after the ICU transfer to the hospital ward	Specialist emergency response team called to attend to the patient in a life-threatening scenario, possibly due to a medication-related complication, after the ICU patients had transferred to the hospital ward
D	Length of stay and mortality (death)	
71	Mortality rate (within 7 days of patient ICU transfer to a hospital ward)	Patient mortality (death) rate within the period stated, due to complications related to medications. Excludes patients transferred from ICU for palliative care
72	Mortality rate (within 30 days of patient ICU transfer to a hospital ward)	Patient mortality (death) rate within the period stated, due to complications related to medications. Excludes patients transferred from ICU for palliative care

E-prescribing, electronic prescribing; GP, general practice.

## Discussion

### Statement of principal findings

This is the first consensus process that focused on identifying priorities for medication-related intervention components and outcome measures to improve medication safety for ICU patients transferring to a hospital ward. The consensus was achieved for >70 items across ICU, hospital ward and public representative panels. The complexity of this medication safety process is underlined by the substantial number of intervention component items deemed important. The participant comments enriched the intervention component data by providing barriers and facilitators, which add to our understanding of system review and refinements required. Finally, the medication-related and patient outcome measures set provide a basis to develop and refine the intervention package prior to testing in future interventional studies.

### Interpretation within the context of the wider literature

Several interventions improve safe and effective medicines use in ICU patients, including electronic prescribing systems (with clinical decision support), clinical pharmacists, changes in staff work schedules, education of staff, clinical guidelines and medicines reconciliation [20–22]. Our findings concur with these broad interventions, adding new detail specific to patient medication safety interventions, processes and important resources when transferring from ICU. De Grood *et al.* [23] achieved ICU and hospital ward staff consensus on the essential content of ICU to hospital ward transfer summaries. The medications section included pre-admission medication, active medications on transfer, the rationale for the medication regimen and any intravenous infusions. Transfer reports should include medicines reconciliation and updated medication prescriptions (orders). We similarly identified the importance of the medication transfer report and multiprofessional handovers (medical, nursing and pharmacy) to hospital ward colleagues. The medication transfer report was identified as a facilitator to medication plan and communication clarity. Our Delphi results expand on recommended medication-related information that requires documentation and communication for ICU patients on hospital ward transfer [8], by identifying the importance of including the rationale for any medication changes.

The merits of medicines reconciliation and review in improving medication safety and outcomes are established for patients in care transitions [7]. Although the timing and delivery of medicines reconciliation and review for ICU patients upon transfer to the hospital ward have been variable [9], we are now able to provide more information on the operationalization of these medication optimization processes, particularly the importance of medication review, within the recommended daily ICU multiprofessional ward round [24, 25]. Furthermore, the importance of easily accessible medicines-related information to facilitate these processes and ultimately minimize process inefficiencies. We have previously identified the importance of staff education and guidelines in reducing the risks of continuing inappropriate medication at the point of hospital discharge [9]. We can now expand on the detail of this in terms of content and scope for both education (ICU and hospital ward staff) and guidelines or protocols’ content and remit. Referrals of high-risk patients to the hospital ward team, specialist teams (e.g. diabetic team) and the primary care team were all deemed important. To effectively operationalize this patient risk stratification, simple evidence-based criteria will be needed for the required staff education and guidelines and be based on multimorbidity, polypharmacy [26] and high-risk medication for errors [27]. Indeed, for older patients being discharged from the hospital, such a prediction tool for medication-related harm has already been proposed [28].

The substantial number of intervention components agreed presents obvious challenges for effective implementation in any evaluation of a complex intervention [12]. It is important to minimize any redundancy within the intervention package, avoiding unnecessary duplication of work (e.g. multiple medicines reconciliations), as identified in the barriers to intervention delivery. Nevertheless, the complexity of hospital care and interfaces means there is often duplication of work and related inefficiencies for health-care professionals and potential inconvenience for patients. Organizational challenges can enforce the adaptation of interventions and compromise intervention fidelity in large-scale quality improvement endeavours [29]. Here, we are able to provide direction to support the implementation of a medication-related intervention package. First, we identify the importance of situational awareness of the multiprofessional ICU care team around the decision to transfer a patient to the hospital ward. Team organizational requirements around own roles and responsibilities as well as other team members around medication safety on patient transfer are explicit and an important intervention facilitator. Finally, we identify important staff, technology and practice scope resources that will support the organizational delivery of an intervention package. Limitations in staff resources in the outreach team and clinical pharmacists were an identified barrier to improving medication safety, consistent with international workforce challenges for ICU clinical pharmacists, including 7-day services [30–32].

Some of our findings differed from the broader transitions in care medication safety recommendations or ICU practice. Patient engagement is acknowledged as a key factor in improving medication safety in transfers of care [7], and more generally, discharge education of patients and family members is an important facilitator to high-quality transfers from ICU [33]. In contrast, we found only the public panel agreed on the importance of medication information intervention components, while there was cross-panel agreement that patient and/or family satisfaction was an important medication outcome to measure. This disparity highlights the importance of understanding how to appropriately engage with ICU patients and their families in medication management as their recovery progresses [34].

Follow-up clinics are seen as a way to support the aftercare of ICU patients and improve post-intensive care syndrome management [35]. We did not have cross-panel consensus on the importance of follow-up clinics for medication safety, with being too late for timely medication review cited as an important barrier.

Patient outcomes are infrequently reported in medication safety studies of ICU patient transfers [9]. Malmgrem *et al.* [36] recently identified the long-term health-related quality of life (HRQoL) patient outcomes for ICU survivors. Our ward panel did not agree that HRQoL was an important patient outcome measure, indicating that further work on staff awareness of the relationship between medication safety and avoidable patient harm is required [37]. There are clear relationships between clinical frailty, multimorbidity and polypharmacy in ICU patients and outcomes [38, 39]. The importance of clinical frailty in older patients on ICU admission was identified, but clinical frailty progression was not regarded as an important patient outcome measure.

### Implications for policy, practice and research

Future work is needed to understand the relative importance of intervention components, and underlining treatment theory, in order to further refine and format into an intervention package suitable for feasibility testing [11]. Further consideration of prioritization and context is also needed to make the package routinely deliverable in different health-care settings [11, 34]. The medication-related and patient outcomes provide an informed outcome measure set to develop and refine an intervention package prior to future evaluation. Finally, the use of a specific HRQoL measure for ICU survivors [36] may be important in a health economic evaluation of a future interventional study.

### Strengths and limitations

There are several strengths to the Delphi process. First, we included a public panel, engaging with intensive care and emergency care patient groups, ensuring that views of participants with a lived experience of critical illness were included. Participant engagement across all phases was high, likely to be aided by the informed consenting process and participant motivation for the subject area, despite being conducted during the coronavirus disease 2019 pandemic. Furthermore, the low attrition rate meant attrition bias was unlikely to be a crucial factor in the results. The richness of participant comments provided an opportunity to explore perceived barriers and facilitators to intervention components. In Phase 3, we provided participants with numerical and graphical representations of all three-panel medians. This method has been reported to reduce intra-panel variability between professionals and public participants [40]. Importantly, we defined the consensus standards *a priori*, using the grading criteria based on the recommendations from the COMET initiative [18]. In terms of potential limitations, study participants were drawn from developed countries, which may limit the generalizability of the findings in less developed countries. Clinical pharmacists comprised the single largest health-care professional group despite extensive efforts to capture multiprofessional stakeholder views. This imbalance in professionally diverse views may present a limitation, although most researchers of medication-related interventional studies in this field are pharmacists [9]. Nevertheless, participant specialty has been reported to influence outcomes in Delphi processes [41].

## Conclusions

Improving medication safety for ICU patients transferring to a hospital ward is complex. Through a Delphi consensus process, including ICU, hospital ward and public panels, we identified many important intervention components. Further work is required to understand how best to engage with ICU patients and/or their families in medication safety on ICU transfer. The intervention development process will be aided by the barriers and facilitators identified in the intervention components. The identified important medication and patient outcomes to measure will inform the design of a future clinical trial.

## Supplementary Material

mzac082_Supp

## Data Availability

The data underlying this article will be shared on reasonable request to the corresponding author.
