# Supplementary material for: A Delphi consensus study to identify priorities for improving and measuring medication safety for intensive care patients on transfer to a hospital ward
Source: Int J Qual Health Care. 2022 Oct 7;34(4):mzac082. doi: 10.1093/intqhc/mzac082 (PMC9633976; doi:10.1093/intqhc/mzac082)
Supplement: mzac082_Supp [file mzac082_supp.zip › suppl_data/Supplementary file 1 IJQHC 27.9.22.docx]

# Supplementary File

| Category | Barrier | Count (%) | Facilitator | Count (%) |
| --- | --- | --- | --- | --- |
| Care Team | Patient/ family as co-agents | 9 (4.4) | Patient/ family as agents | 18 (8.8) |
|  | Limitations in staff knowledge and understanding of medication | 5 (2.4) | Staff knowledge and understanding of medication | 9 (4.4) |
|  | Assumption somebody else will review medicines | 3 (1.5) | Patient/ family knowledge and understanding of medication | 3 (1.5) |
|  | Too much information for patient/ family | 3 (1.5) |  |  |
|  | Too many staff involved | 2 (1) |  |  |
|  | Patient/ family lack of knowledge and understanding of medication | 1 (0.5) |  |  |
| Organisational Conditions | Lack of staff resources (e.g. outreach team, pharmacists) | 10 (4.9) | Clear staff roles and responsibilities | 22 (10.7) |
|  | Follow up clinics conducted too late for timely medication review | 9 (4.4) | Multiprofessional involvement in medication review | 7 (3.4) |
|  | Excessive staff workload | 3 (1.5) | Onus on ICU staff for medicines reconciliation/ medication review pre-transfer | 6 (2.9) |
|  | Lack of senior healthcare staff medication review on hospital ward | 3 (1.5) | Easy access to staff who are prescribers | 1 (0.5) |
|  | Poor patient transfer planning | 2 (1) | Staff time for professional support activities (e.g. guidelines) | 1 (0.5) |
|  | High turnover of staff (e.g. rotations) | 1 (0.5) | Time available to plan transfer fully | 1 (0.5) |
| Tools and Technologies | Checklist burden & fatigue | 6 (2.9) | Medicines-related information easily accessible | 16 (7.8) |
|  | Checklist too generic/ non-specific | 3 (1.5) | Automated systems to support key processes | 8 (3.9) |
|  | Non-integrated e-prescribing systems | 1 (0.5) | Integrated e-prescribing systems included ICU, hospital ward and primary care services | 1 (0.5) |
|  | Over-reliance on e-prescribing systems | 1 (0.5) | Mandatory transfer processes | 1 (0.5) |
|  | Too many guidelines | 1 (0.5) |  |  |
| Tasks | Duplication of work | 10 (4.9) | Clear medication plan and communication | 15 (7.3) |
|  | ICU medication complex or high-risk | 9 (4.4) | Medicines reconciliation at each transfer stage | 1 (0.5) |
|  | Complexity of ICU patients | 2 (1) | Re-prescribe all medication prior to ICU transfer | 1 (0.5) |
|  | Frequency of medication information changing | 1 (0.5) | Medication review after hospital discharge | 2 (1) |
|  | Lack of medication information communicated | 1 (0.5) |  |  |
|  | Over reliance on medicines reconciliation at single time-point | 1 (0.5) |  |  |
| External Environment | Transfer time pressures or uncertainties | 3 (1.5) |  |  |
|  | Limited healthcare resources | 1 (0.5) |  |  |
|  | Variation in community pharmacy use by patients/family | 1 (0.5) |  |  |

**Table S1. Socio-technical categorisation of barriers and facilitators to medication safety identified from participant comments in Phase One.**

| Item | Panel not reaching consensus | Panel grade in Phase 3, median (IQR) | Panel % “Important” | Panel % “Unimportant” |
| --- | --- | --- | --- | --- |
| All appropriate ICU clinical staff are aware of the approximate time the patient is expected to be transferred to the hospital ward | Ward | 7 (6,8) | 65.0 | 0.0 |
| All appropriate ICU clinical staff aware of which clinical specialty the patient's care is being transferred to | ICU | 7 (6,8) | 57.7 | 0.0 |
| ICU follow up clinic provided after the patient (high-risk) hospital discharge with medication review component | Ward | 6 (6,7) | 36.8 | 5.3 |
| High-risk patients for medication-related problems (e.g. medication continuity or transfer errors) identified and planned/referred for follow up by ICU Outreach Team | ICU | 7 (6,7) | 65.7 | 3.0 |
| High-risk patients for medication-related problems (e.g. medication continuity or transfer errors) identified and planned/referred for follow up by ICU Follow-up clinic (usually conducted 1-3 months after hospital transfer | Ward | 6 (4,7) | 31.6 | 15.8 |
| Timing of patient adverse drug events on ward after ICU transfer | ICU | 6 (5,7) | 35.2 | 2.8 |
| Clinical frailty scale on ICU transfer (older people only, 65+ years) | ICU | 7 (6,7) | 69.6 | 1.4 |
| Quality of Life measures | Ward | 7 (6,8) | 55.6 | 16.7 |
| Readmission rate (unplanned) to hospital (30 days) | Ward | 7 (6,8) | 52.6 | 0.0 |
| Length of patient hospital stay post-ICU transfer | ICU | 7 (6,8) | 69.0 | 0.0 |

**Table S2. Items meeting important criteria for two panels only.**
