# Supplementary material for: A Delphi consensus study to identify priorities for improving and measuring medication safety for intensive care patients on transfer to a hospital ward
Source: Int J Qual Health Care. 2022 Oct 7;34(4):mzac082. doi: 10.1093/intqhc/mzac082 (PMC9633976; doi:10.1093/intqhc/mzac082)
Supplement: mzac082_Supp [file mzac082_supp.zip › suppl_data/Supplementary file 2 IJQHC.pdf]

Section	Subsection	Item	ICU Panel Phase One Median (IQR)	"Important" %	"Not-Important" %	Phase Two Median (IQR)	"Important" %	"Not-Important" %	Phase Three Median (IQR)	"Important" %	"Not-Important" %	WARD Panel Phase One Median (IQR)	"Important" %	"Not-Important" %	Phase Two Median (IQR)	"Important" %	"Not-Important" %	Phase Three Median (IQR)	"Important" %	"Not-Important" %	PUBLIC Panel Phase One Median (IQR)	"Important" %	"Not-Important" %	Phase Two Median (IQR)	"Important" %	"Not-Important" %	Phase Three Median (IQR)	"Important" %	"Not-Important" %
1	A	All appropriate ICU clinical staff are aware of the decision that the patient is deemed ready for transfer from ICU to a hospital ward	8 (7,9)	88.9	1.2	8.5 (8,9)	96.1	0.0	N/A	N/A	N/A	9 (7,9)	82.4	0.0	9 (8,9)	94.7	0.0	N/A	N/A	N/A	8 (7.25,9)	95.0	0.0	9 (8,9)	100.0	0.0	N/A	N/A	N/A
		All appropriate ICU clinical staff are aware of the approximate time the patient is expected to be transferred to the hospital ward	7 (6,8)	63.4	2.4	7 (7,8)	76.3	1.3	7 (7,8)	84.5	1.4	7 (6,8)	63.2	5.3	7 (6,8)	63.2	0.0	7 (6,8)	65.0	0.0	7 (7,9)	84.2	0.0	8 (7,9)	90.0	0.0	8 (7,9)	94.7	0.0
		All ICU clinical staff are aware of their own specific roles and responsibilities in the safety and continuity of medication in ICU patients on transfer to a hospital ward	9 (8,9)	93.7	0.0	9 (9,9)	100.0	0.0	N/A	N/A	N/A	8 (8,9)	88.2	0.0	8 (8,9)	89.5	0.0	N/A	N/A	N/A	9 (8,9)	95.0	0.0	9 (8,9)	100.0	0.0	N/A	N/A	N/A
		All ICU clinical staff are aware of the specific roles and responsibilities of other healthcare professionals in the safety and continuity of medication in ICU patients upon transfer to a hospital ward	8 (7,9)	81.5	2.5	8 (7,8)	89.6	0.0	N/A	N/A	N/A	7 (6,8)	73.7	0.0	7 (7,8)	80.0	0.0	N/A	N/A	N/A	8 (7,9)	90.0	0.0	8 (7,9)	95.0	0.0	N/A	N/A	N/A
		Multiprofessional ward rounds are undertaken daily on ICU with attendance by all appropriate healthcare professions involved in patient medication review	9 (8,9)	93.6	1.3	9 (9,9)	97.4	0.0	N/A	N/A	N/A	9 (8,9)	95.0	0.0	9 (8.25,9)	95.0	0.0	N/A	N/A	N/A	9 (8,9)	94.4	0.0	9 (8,9)	100.0	0.0	N/A	N/A	N/A
		Education of ICU staff on medication at high-risk for inappropriate continuation on transfer to a hospital ward (e.g. stress ulceration prophylaxis, antipsychotics for delirium, antiarrhythmics, bronchodilators)	8 (7,9)	89.7	0.0	9 (8,9)	100.0	0.0	N/A	N/A	N/A	8 (7,9)	80.0	0.0	8 (7.25,9)	100.0	0.0	N/A	N/A	N/A	9 (8,9)	94.7	0.0	9 (9,9)	100.0	0.0	N/A	N/A	N/A
		Education of ICU staff on medication at high-risk for failure to restart on transfer to a hospital ward (e.g. antiplatelets, anticoagulants, mental health medication, cardiovascular medication)	8 (7.75,9)	93.6	0.0	8.5 (8,9)	98.7	0.0	N/A	N/A	N/A	8 (7,9)	90.0	0.0	8 (8,9)	95.0	0.0	N/A	N/A	N/A	9 (8,9)	100.0	0.0	9 (9,9)	100.0	0.0	N/A	N/A	N/A
		Education of hospital ward staff on medication at high-risk for inappropriate continuation on transfer to a hospital ward (e.g. stress ulceration prophylaxis, antipsychotics for delirium, bronchodilators)	8 (7,9)	81.0	0.0	8 (7,9)	96.1	0.0	N/A	N/A	N/A	8 (7,8.75)	80.0	0.0	8 (7,9)	90.0	0.0	N/A	N/A	N/A	9 (8,9)	94.4	0.0	9 (9,9)	100.0	0.0	N/A	N/A	N/A
		Education of hospital ward staff on medication at high-risk for failure to restart on transfer to a hospital ward (e.g. antiplatelets, anticoagulants, mental health medication, cardiovascular medication)	8 (7,9)	87.2	0.0	8 (8,9)	100.0	0.0	N/A	N/A	N/A	8 (7,9)	90.0	0.0	8 (8,9)	95.0	0.0	N/A	N/A	N/A	9 (8,9)	100.0	0.0	9 (9,9)	100.0	0.0	N/A	N/A	N/A
		All appropriate ICU clinical staff aware of which clinical specialty the patient's care is being transferred to	N/A	N/A	N/A	6 (5,8)	49.3	5.3	7 (6,8)	57.7	0.0	N/A	N/A	N/A	7 (6,8)	70.0	0.0	7 (6,8)	70.0	0.0	N/A	N/A	N/A	9 (8,9)	94.1	0.0	9 (8,9)	94.1	0.0
	B	Guidelines (ICU/ hospital) on short-term ICU medication, including indication and when to stop or wean off	7 (6,8)	72.0	2.4	7 (7,8)	82.9	1.3	N/A	N/A	N/A	8 (6.25,9)	75.0	0.0	9 (8,9)	90.0	0.0	N/A	N/A	N/A	8 (7,9)	90.0	0.0	8 (8,9)	100.0	0.0	N/A	N/A	N/A
		Medication checklist on ICU patient transfer to a hospital ward (is recommended)	7 (6,8)	65.0	3.8	7 (6,8)	72.0	2.7	N/A	N/A	N/A	8 (7,9)	78.9	5.3	8 (8,9)	90.0	0.0	N/A	N/A	N/A	9 (7,9)	90.0	0.0	9 (8,9)	100.0	0.0	N/A	N/A	N/A
		Medication checklist on ICU patient transfer to a hospital ward (is mandatory)	7 (5,8)	58.2	2.5	7 (6,8)	64.0	2.7	8 (7,9)	81.4	0.0	7 (6,8)	57.9	5.3	8 (6,8)	70.0	5.0	8 (7,9)	85.0	0.0	9 (9,9)	100.0	0.0	9 (9,9)	100.0	0.0	N/A	N/A	N/A
		ICU to hospital ward transfer protocol with medication section	7 (6,9)	70.9	5.1	7 (7,8.25)	86.5	2.7	N/A	N/A	N/A	8 (6.25,9)	75.0	0.0	8 (8,9)	85.0	0.0	N/A	N/A	N/A	9 (8,9)	94.7	0.0	9 (9,9)	100.0	0.0	9 (9,9)	100.0	0.0
		Hospital ward admission protocol with ICU patient step-down medication review component	7 (5,8)	61.3	5.0	7 (6,8)	74.7	4.0	N/A	N/A	N/A	7.5 (6,8)	70.0	10.0	8 (7,8)	90.0	5.0	N/A	N/A	N/A	8 (7,9)	100.0	0.0	8 (8,9)	100.0	0.0	N/A	N/A	N/A
		Hospital patient medication discharge protocol	7 (6,9)	72.2	2.5	7 (7,9)	84.2	1.3	N/A	N/A	N/A	7 (6,9)	63.2	5.3	7 (7,8.75)	80.0	0.0	N/A	N/A	N/A	9 (8,9)	100.0	0.0	9 (9,9)	100.0	0.0	N/A	N/A	N/A
		Guideline on identification of high-risk patients or medications	7 (6,8.5)	72.8	0.0	7 (7,8)	86.8	1.3	N/A	N/A	N/A	7 (6,8)	65.0	10.0	7 (6.25,8)	75.0	5.0	N/A	N/A	N/A	9 (8,9)	100.0	0.0	9 (9,9)	100.0	0.0	N/A	N/A	N/A
	C	Medication (short-term indication), documentation of criteria to stop or wean	8 (7,9)	89.9	1.3	9 (8,9)	98.7	0.0	N/A	N/A	N/A	8 (7.75,9)	88.9	0.0	9 (8,9)	95.0	0.0	N/A	N/A	N/A	8 (7,9)	89.5	0.0	9 (7,9)	94.4	0.0	N/A	N/A	N/A
		Medication (chronic/ long-term), documentation of criteria to restart/re-titrate	8 (7,9)	82.5	1.3	8 (7,9)	97.4	0.0	N/A	N/A	N/A	8 (7,9)	89.5	0.0	9 (7.25,9)	95.0	0.0	N/A	N/A	N/A	8.5 (7,9)	88.9	0.0	9 (8,9)	100.0	0.0	N/A	N/A	N/A
		Medication intended to continue are documented	8 (7,9)	77.5	2.5	8 (8,9)	90.8	0.0	N/A	N/A	N/A	9 (8,9)	84.2	0.0	9 (9,9)	90.0	0.0	N/A	N/A	N/A	9 (7.25,9)	95.0	0.0	9 (8.75,9)	100.0	0.0	N/A	N/A	N/A
		Medication route changes are documented	7 (5,8)	59.3	3.7	7 (6,7.75)	68.4	2.6	7 (7,9)	80.3	0.0	7 (6,8)	57.9	10.5	7.5 (6,8)	60.0	5.0	7 (6,8)	73.7	0.0	9 (7,9)	94.7	0.0	9 (9,9)	100.0	0.0	9 (9,9)	100.0	0.0
		Medication dose changes and reasons are documented	8 (7,9)	77.8	0.0	8 (8,9)	97.4	0.0	N/A	N/A	N/A	8 (7,9)	84.2	0.0	8 (8,9)	95.0	0.0	N/A	N/A	N/A	9 (7.25,9)	90.0	0.0	9 (9,9)	100.0	0.0	N/A	N/A	N/A
		Medication permanently discontinued and reasons are documented	9 (8,9)	94.9	0.0	9 (9,9)	100.0	0.0	N/A	N/A	N/A	9 (7,9)	88.9	0.0	9 (9,9)	95.0	0.0	N/A	N/A	N/A	9 (7.25,9)	95.0	0.0	9 (8.5,9)	94.4	0.0	N/A	N/A	N/A
		Medication temporarily held/omitted and reasons are documented	8 (7,9)	88.8	1.3	9 (8,9)	100.0	0.0	N/A	N/A	N/A	8.5 (8,9)	94.4	0.0	9 (8.25,9)	95.0	0.0	N/A	N/A	N/A	9 (8,9)	90.0	0.0	9 (9,9)	94.7	0.0	N/A	N/A	N/A
		Re-prescribe all medication on ICU prior to hospital ward transfer	5.5 (3,7)	30.8	28.2	6 (3.75,6.25)	24.3	24.3	6 (5,8)	35.3	14.7	6.5 (5.25,9)	50.0	10.0	8 (6,9)	65.0	10.0	7 (6,9)	63.2	10.5	9 (7.25,9)	87.5	0.0	9 (9,9)	82.4	0.0	9 (9,9)	94.1	0.0
		Medicines reconciliation on admission to ICU	9 (8,9)	91.0	0.0	9 (9,9)	97.3	0.0	N/A	N/A	N/A	8 (7,9)	89.5	0.0	9 (8,9)	89.5	0.0	N/A	N/A	N/A	8 (6.75,9)	77.8	0.0	8.5 (7,9)	94.4	0.0	N/A	N/A	N/A
		Medicines reconciliation on ICU prior to transfer to the hospital ward	8 (7,9)	77.2	0.0	9 (8,9)	90.7	0.0	N/A	N/A	N/A	7 (6,8.75)	70.0	5.0	7 (7,8.75)	85.0	0.0	N/A	N/A	N/A	8 (7,9)	89.5	0.0	8 (7,9)	94.4	0.0	N/A	N/A	N/A
		Medicines reconciliation on the hospital ward after ICU to hospital ward transfer	8 (6.5,9)	75.3	3.7	8 (7,9)	86.7	1.3	N/A	N/A	N/A	7 (6,8)	70.0	5.0	7.5 (7,8)	85.0	5.0	N/A	N/A	N/A	8 (7,9)	88.9	0.0	8 (7,9)	94.1	0.0	N/A	N/A	N/A
		Medicines reconciliation prior to discharge from the hospital	9 (8,9)	91.3	2.5	9 (9,9)	100.0	0.0	N/A	N/A	N/A	7.5 (5,9)	65.0	5.0	8 (7,9)	80.0	0.0	N/A	N/A	N/A	9 (7,9)	84.2	0.0	9 (8,9)	94.4	0.0	N/A	N/A	N/A
		Medicines reconciliation in GP practice after discharge from the hospital	8 (7,9)	77.9	2.6	8 (7,9)	90.4	1.4	N/A	N/A	N/A	8.5 (7,9)	80.0	0.0	9 (8,9)	85.0	0.0	N/A	N/A	N/A	9 (7,9)	94.7	5.3	9 (7,9)	94.4	5.6	N/A	N/A	N/A
		Medicines reconciliation in Community Pharmacy after discharge from the hospital	7 (5,9)	54.7	10.7	7 (5,9)	57.1	8.6	7 (6,8)	61.5	6.2	6.5 (4,7)	50.0	10.0	7 (4,7)	57.9	10.5	7 (5.75,7)	61.1	5.6	7 (5,9)	63.2	0.0	7 (7,9)	81.3	0.0	7 (7,8.75)	87.5	0.0
		Medication review - undertaken daily on ICU	9 (8,9)	93.5	0.0	9 (9,9)	100.0	0.0	N/A	N/A	N/A	8 (6,9)	73.7	0.0	9 (8,9)	95.0	0.0	N/A	N/A	N/A	8 (8,9)	78.9	0.0	9 (8,9)	94.1	0.0	N/A	N/A	N/A
		Multiprofessional ICU ward round includes patient medication review – undertaken daily on ICU	9 (8,9)	96.2	0.0	9 (9,9)	98.7	0.0	N/A	N/A	N/A	8 (7,9)	84.2	0.0	9 (8.25,9)	100.0	0.0	N/A	N/A	N/A	9 (7.25,9)	85.0	0.0	9 (8,9)	89.5	0.0	N/A	N/A	N/A
		Medication review – on ICU prior to transfer to a hospital ward	9 (8,9)	88.8	0.0	9 (9,9)	100.0	0.0	N/A	N/A	N/A	8 (7,9)	95.0	0.0	8 (7.25,9)	100.0	0.0	N/A	N/A	N/A	8 (7.25,9)	95.0	0.0	9 (8,9)	100.0	0.0	N/A	N/A	N/A
		Medication review on the hospital ward soon after transfer to a hospital ward	8 (7,9)	81.3	1.3	8 (8,9)	92.0	1.3	N/A	N/A	N/A	7.5 (7,9)	85.0	0.0	8 (7.25,9)	95.0	0.0	N/A	N/A	N/A	8 (7,9)	95.0	0.0	8.5 (7,9)	100.0	0.0	N/A	N/A	N/A
	D	Electronic prescribing systems in ICU and hospital ward are fully integrated	9 (7,9)	88.5	2.6	9 (9,9)	94.4	0.0	N/A	N/A	N/A	9 (6.75,9)	77.8	0.0	9 (9,9)	100.0	0.0	N/A	N/A	N/A	8 (8,9)	100.0	0.0	8.5 (8,9)	100.0	0.0	N/A	N/A	N/A
		ICU clinical pharmacy services provided 7-days per week	8 (7,9)	79.7	0.0	8 (8,9)	87.7	0.0	N/A	N/A	N/A	8 (7,9)	83.3	5.6	9 (8,9)	90.0	0.0	N/A	N/A	N/A	9 (8,9)	95.0	0.0	9 (9,9)	94.4	0.0	N/A	N/A	N/A
		ICU outreach team follow up of ICU patients on transfer to a hospital ward include a medication review component	7 (6,8)	59.5	6.3	7 (6,7)	63.4	5.6	7 (7,8)	85.3	2.9	7 (5,8)	55.0	0.0	7 (6.25,8)	75.0	0.0	7 (7,8)	100.0	0.0	8 (7.25,9)	95.0	0.0	8.5 (7.75,9)	100.0	0.0	9 (8,9)	100.0	0.0
		ICU follow up clinic provided after the patient (high-risk) hospital discharge with medication review component	7 (6,8)	70.5	7.7	7 (7,8)	76.1	4.2	7 (7,9)	79.7	1.4	6 (4,7)	30.0	10.0	6 (5,6.75)	25.0	5.0	6 (6,7)	36.8	5.3	8 (7,9)	89.5	0.0	9 (7,9)	100.0	0.0	9 (7.75,9)	100.0	0.0
		Healthcare professionals involved in the medication review of ICU patient have appropriate prescribing authority to their role	7 (6,9)	67.5	2.5	7 (7,9)	83.6	1.4	N/A	N/A	N/A	7 (5,8)	55.0	0.0	7 (6,7.75)	70.0	0.0	N/A	N/A	N/A	8 (7,9)	83.3	0.0	7.5 (7,9)	93.8	0.0	N/A	N/A	N/A
	E	High-risk patients for medication-related problems (e.g. medication continuity or transfer errors) identified and planned/referred for follow up by ICU Outreach Team	6 (5,7)	47.4	9.0	6 (5,7)	44.3	5.7	7 (6,7)	65.7	3.0	7 (6,8)	65.0	0.0	7 (6,8)	65.0	0.0	7 (7,8)	89.5	0.0	8 (7,9)	94.1	0.0	8 (7,9)	100.0	0.0	8 (7,9)	100.0	0.0
		High-risk patients for medication-related problems (e.g. medication continuity or transfer errors) identified and planned/referred for follow up by the hospital Ward Team	7 (6,8)	72.5	1.3	7 (7,8)	86.3	1.4	N/A	N/A	N/A	8 (6,8.75)	70.0	0.0	8 (8,9)	85.0	0.0	N/A	N/A	N/A	8 (7,9)	89.5	0.0	8 (7,9)	100.0	0.0	N/A	N/A	N/A
		High-risk patients for medication-related problems (e.g. medication continuity or transfer errors) identified and planned/referred for follow up by GP Practice	7 (5,8)	54.3	6.2	7 (5,8)	58.3	1.4	7 (7,8)	77.1	0.0	8 (6,8)	70.0	15.0	7 (7,8)	85.0	5.0	7 (7,8)	84.2	5.3	7 (7,9)	84.2	0.0	8 (7,9)	94.4	0.0	8 (7,9)	94.7	0.0
		High-risk patients for medication-related problems (e.g. medication continuity or transfer errors) identified and planned/referred for follow up by Community Pharmacist	6 (4,8)	44.9	14.1	6 (4,7)	40.6	7.2	6 (5,7)	39.7	4.8	6.5 (5.25,7.75)	50.0	15.0	6 (6,7)	47.4	10.5	6 (6,7.25)	27.8	5.6	7 (6,9)	57.9	0.0	7 (6,8)	64.7	0.0	7 (7,8)	78.9	0.0
		High-risk patients for medication-related problems (e.g. medication continuity or transfer errors) identified and planned/referred for follow up by ICU Follow-up clinic (usually conducted 1-3 months after hospital transfer)	7 (5,8)	53.2	11.4	7 (5,8)	62.5	8.3	7 (6,8)	70.6	5.9	4.5 (3.25,6)	15.0	25.0	6 (4,6.75)	25.0	15.0	6 (4,7)	31.6	15.8	8 (7,9)	84.2	5.3	8 (7,9)	88.9	0.0	8 (7,9)	94.7	0.0
		Referral to specialist team for medication review (e.g. diabetes, care of the elderly, psychiatry, cardiology, acute pain teams)	7 (6,8)	63.9	1.2	7 (6,8)	73.0	0.0	N/A	N/A	N/A	7 (7,8)	80.0	0.0	7 (7,8)	90.0	0.0	N/A	N/A	N/A	9 (8.25,9)	100.0	0.0	9 (9,9)	100.0	0.0	N/A	N/A	N/A
	F	Antimicrobial(s) indication(s) included	9 (8,9)	96.2	0.0	9 (9,9)	100.0	0.0	N/A	N/A	N/A	9 (8,9)	94.7	0.0	9 (8,9)	100.0	0.0	N/A	N/A	N/A	8 (7,9)	92.9	0.0	8 (7,9)	100.0	0.0	N/A	N/A	N/A
		Antimicrobial start dates included	9 (7,9)	92.4	0.0	9 (9,9)	98.6	0.0	N/A	N/A	N/A	9 (7,9)	84.2	0.0	9 (8,9)	95.0	0.0	N/A	N/A	N/A	8 (7,9)	92.9	0.0	8 (7,9)	100.0	0.0	N/A	N/A	N/A
		Antimicrobial review dates included	8 (7,9)	87.3	0.0	9 (8,9)	95.9	0.0	N/A	N/A	N/A	8 (7,9)	78.9	0.0	9 (8,9)	90.0	0.0	N/A	N/A	N/A	8 (7,9)	92.9	0.0	9 (7,9)	100.0	0.0	N/A	N/A	N/A
		Medication report for the ICU patient on transfer to a hospital ward to hospital ward staff	8 (6,9)	70.5	3.8	8 (7,9)	84.7	0.0	N/A	N/A	N/A	8 (7,9)	84.2	0.0	9 (8,9)	90.0	0.0	N/A	N/A	N/A	9 (8,9)	100.0	0.0	9 (8.75,9)	100.0	0.0	N/A	N/A	N/A
		Medication report for the ICU patient on transfer to a hospital ward to GP practice staff	6 (4,8)	48.1	24.1	6 (4,7)	35.2	23.9	6 (4,7)	36.2	21.7	4 (2,7)	36.8	42.1	4.5 (2,7)	30.0	45.0	4 (2,7)	31.6	42.1	8.5 (7,9)	90.0	0.0	9 (7.75,9)	94.4	0.0	9 (8,9)	100.0	0.0
		Medication report for the ICU patient on transfer to a hospital ward to community pharmacist	5 (3,7)	31.2	32.5	5 (3,6)	19.7	35.2	4.5 (3,6)	15.2	33.3	4 (2,5.75)	15.0	45.0	4 (1,4)	5.3	47.4	3 (1,4)	5.6	55.6	8 (6,9)	68.4	5.3	7.5 (4.5,8)	68.8	12.5	8 (5.5,8.25)	66.7	5.6
		Medication report for the ICU patient on transfer to a hospital ward for the patient or family	6 (4,7)	38.0	24.1	6 (4,7)	29.6	21.1	5 (4,6.5)	24.6	20.3	4.5 (3.25,6)	20.0	25.0	5 (4,7)	30.0	10.0	4 (4,7)	26.3	15.8	8.5 (7,9)	85.0	0.0	9 (7,9)	84.2	0.0	9 (8,9)	94.4	0.0
		Handover (verbal) of medication therapy information and review requirements by ICU medical team to hospital ward medical team	7 (5,8)	58.5	6.1	7 (6,8)	71.6	2.7	N/A	N/A	N/A	7.5 (6,8.75)	65.0	10.0	8 (6.25,8)	75.0	0.0	N/A	N/A	N/A	8 (6,9)	73.7	5.3	8 (7,9)	83.3	0.0	N/A	N/A	N/A
		Handover (verbal) of medication therapy information, review and administration requirements by ICU nursing team to hospital ward nursing team	7 (5,8)	59.8	3.7	7 (6,8)	74.3	0.0	N/A	N/A	N/A	7 (6,8)	57.9	0.0	7 (6.25,8.75)	75.0	0.0	N/A	N/A	N/A	8 (6,9)	73.7	5.3	9 (8,9)	88.9	0.0	N/A	N/A	N/A
		Handover (verbal or electronic) of medication therapy information and review requirements by ICU pharmacy team to ward pharmacy team	8 (7,9)	79.0	0.0	8 (7.75,9)	86.5	0.0	N/A	N/A	N/A	9 (6.25,9)	75.0	5.0	9 (7.25,9)	85.0	0.0	N/A	N/A	N/A	8 (7,9)	89.5	0.0	8 (8,9)	94.1	0.0	N/A	N/A	N/A
		Patient and/or family provided with a verbal summary of any ICU medication changes and plans for medication review on the hospital ward	5 (3,7)	31.3	26.5	5 (4,6)	17.6	23.0	5 (4,6)	20.0	15.7	6 (4,7)	35.0	20.0	6 (4,7)	40.0	10.0	6 (4,7)	42.1	5.3	8 (7,9)	85.0	5.0	8 (6.75,9)	77.8	5.6	8 (7,9)	78.9	5.3
		Patient and/or family provided with a written summary of any ICU medication changes and plans for medication review on the hospital ward	5 (3,6.5)	24.7	34.6	5 (3,5.5)	13.7	32.9	5 (4,6)	20.0	22.9	4.5 (3,6)	20.0	30.0	5.5 (3.25,6.75)	25.0	25.0	5 (3,7)	26.3	26.3	9 (7,9)	90.0	5.0	9 (7,9)	88.9	0.0	9 (8,9)	94.7	0.0
		Patient and/or family provided with a leaflet explaining that as part of their ICU care, several medicines may have needed to be changed temporarily or permanently	6 (5,8)	45.0	12.5	6 (5,7)	39.2	6.8	6 (6,7)	48.6	2.9	6 (4,7)	36.8	15.8	6 (5,7)	35.0	10.0	6 (5,7)	36.8	5.3	7 (6,9)	70.0	0.0	7 (7,9)	83.3	0.0	7 (7,9)	84.2	0.0
2	A	Number of changes in medicines on ICU transfer to the hospital ward	5 (4,7)	32.9	20.7	5 (4,7)	25.7	16.2	5 (5,6)	23.9	8.5	6 (5,7.75)	45.0	10.0	6 (5.25,7)	35.0	10.0	6 (5,7)	42.1	10.5	8 (6.75,9)	77.8	5.6	8 (7.25,9)	87.5	6.3	8 (8,9)	88.2	5.9
		Number of changes in high-risk medicines on ICU transfer to the hospital ward	7 (5,8)	56.1	13.4	7 (6,8)	67.6	2.7	7 (7,8)	80.0	0.0	8 (5.25,9)	60.0	5.0	8 (6,9)	70.0	0.0	8 (6,9)	73.7	0.0	8 (7,9)	94.4	5.6	9 (8,9)	93.8	6.3	9 (8,9)	94.1	5.9
		Number of changes in medicines on hospital discharge	7 (5,8)	55.6	11.1	7 (6,8)	70.3	4.1	7 (7,8)	83.1	2.8	6.5 (5,9)	50.0	10.0	7 (5.25,9)	60.0	5.0	7 (6,9)	73.7	0.0	8 (5.75,9)	72.2	5.6	8.5 (7.25,9)	81.3	6.3	9 (8,9)	94.1	5.9
		Number of changes in high-risk medicines on hospital discharge	8 (6,9)	69.1	11.1	8 (7,9)	82.4	5.4	N/A	N/A	N/A	7.5 (6,9)	55.0	10.0	8 (6,9)	70.0	5.0	N/A	N/A	N/A	8.5 (6.5,9)	77.8	5.6	9 (7.25,9)	87.5	6.3	N/A	N/A	N/A
	B	Number of medication errors on ICU transfer to the hospital ward	7 (5,9)	61.7	9.9	7 (6.75,9)	75.7	0.0	N/A	N/A	N/A	7 (6.75,9)	77.8	0.0	7 (7,9)	90.0	0.0	N/A	N/A	N/A	8 (6.75,9)	77.8	0.0	8 (8,9)	88.2	0.0	N/A	N/A	N/A
		Number of medication errors on hospital discharge	7 (5,9)	63.0	11.1	7 (6,9)	71.6	0.0	N/A	N/A	N/A	8 (7,9)	78.9	0.0	8.5 (7,9)	90.0	0.0	N/A	N/A	N/A	8 (7,9)	78.9	0.0	8.5 (7.75,9)	88.9	0.0	N/A	N/A	N/A
		Clinical severity of medication errors on ICU transfer to the hospital ward	8 (6,9)	73.8	6.3	8 (7,9)	87.8	0.0	N/A	N/A	N/A	8 (7,9)	81.3	0.0	8 (8,9)	95.0	0.0	N/A	N/A	N/A	9 (8,9)	100.0	0.0	9 (9,9)	100.0	0.0	N/A	N/A	N/A
		Clinical severity of medication errors on hospital discharge	8 (6,9)	72.5	7.5	8 (7,9)	85.1	0.0	N/A	N/A	N/A	8 (6,9)	70.6	0.0	8 (7,9)	85.0	0.0	N/A	N/A	N/A	9 (8,9)	94.7	0.0	9 (8.75,9)	94.4	0.0	N/A	N/A	N/A
		Number of clinically important "moderate" or higher severity medication errors on ICU transfer to the hospital ward	8 (5,9)	65.4	11.1	8 (7,9)	83.8	0.0	N/A	N/A	N/A	7 (6.5,8)	76.5	0.0	7.5 (7,8.75)	90.0	0.0	N/A	N/A	N/A	8 (7,9)	83.3	0.0	8 (7.75,9)	94.4	0.0	N/A	N/A	N/A

		Number of clinically important "serious" or higher severity medication errors on ICU transfer to the hospital ward	8 (6,9)	72.5	7.5	8 (8,9)	94.6	0.0	N/A	N/A	N/A	8 (6.5,9)	76.5	0.0	9 (8,9)	90.0	0.0	N/A	N/A	N/A	9 (8,9)	94.7	0.0	9 (8,9)	100.0	0.0	N/A	N/A	N/A
		Number of clinically important "moderate" or higher severity medication errors on hospital discharge	8 (5,9)	64.6	11.4	8 (7,9)	82.4	1.4	N/A	N/A	N/A	7 (6,8.25)	66.7	5.6	7.5 (6,8.75)	70.0	0.0	N/A	N/A	N/A	8.5 (7,9)	80.0	0.0	9 (8,9)	94.4	0.0	N/A	N/A	N/A
		Number of clinically important "serious" or higher severity medication errors on hospital discharge	8 (5,9)	67.9	10.3	9 (7.75,9)	86.5	1.4	N/A	N/A	N/A	8 (6,9)	72.2	0.0	8 (7,9)	80.0	0.0	N/A	N/A	N/A	9 (7,9)	90.0	0.0	9 (8,9)	100.0	0.0	N/A	N/A	N/A
		Categorisation of medication errors (e.g. by psychological approach)	7 (6,8)	59.7	7.8	7 (6,7)	66.7	4.2	7 (7,7)	76.5	4.4	7 (6,8)	68.4	5.3	7 (6,8)	73.7	5.3	7 (7,8)	88.9	0.0	8 (7,9)	100.0	0.0	8 (7.25,9)	100.0	0.0	8 (7.5,9)	100.0	0.0
	C	Hospital ward multiprofessional team satisfaction with the transfer medication information and plan (from ICU to ward)	7 (5,8)	56.8	6.2	7 (6,7)	72.6	0.0	N/A	N/A	N/A	7 (6.25,8)	75.0	0.0	7 (7,8)	90.0	0.0	N/A	N/A	N/A	8.5 (7,9)	85.0	0.0	9 (8,9)	94.4	0.0	N/A	N/A	N/A
		GP practice staff satisfaction with medication information and plan (from hospital)	7 (5,8)	56.8	7.4	7 (6,7)	71.2	4.1	N/A	N/A	N/A	7 (6,8.75)	70.0	15.0	7 (7,7.75)	80.0	5.0	N/A	N/A	N/A	8 (7,9)	80.0	0.0	8 (8,9)	88.9	0.0	N/A	N/A	N/A
		Community pharmacist satisfaction with transfer medication information and plan (from hospital)	6 (5,7)	41.8	15.2	6 (5,7)	38.9	9.7	6 (5,7)	42.6	7.4	7 (4,8)	55.0	20.0	7 (4,7)	55.0	20.0	7 (5,7)	63.2	15.8	7 (6,9)	73.7	5.3	7 (7,9)	82.4	5.9	7 (7,9)	83.3	0.0
		Patient and/or family satisfaction with the ICU medication transfer information and plan (from ICU and hospital)	7 (5,9)	64.6	5.1	7 (6,8)	73.0	1.4	N/A	N/A	N/A	7 (6,9)	65.0	0.0	7 (6.25,9)	75.0	5.0	N/A	N/A	N/A	9 (7,9)	80.0	0.0	9 (8,9)	88.9	0.0	N/A	N/A	N/A
		Time to first medication review after ICU patient transfer to a hospital ward	7 (6,9)	60.5	2.5	7 (6,8)	71.2	1.4	N/A	N/A	N/A	7.5 (6.25,9)	75.0	0.0	8 (7,9)	85.0	0.0	N/A	N/A	N/A	7.5 (7,9)	80.0	0.0	8 (7,9)	88.2	0.0	N/A	N/A	N/A
3	A	Adverse drug events on the hospital ward after ICU transfer (number documented for patient)	7 (5,9)	59.5	5.1	7 (7,8.25)	78.4	2.7	N/A	N/A	N/A	7 (6,9)	63.2	10.5	7.5 (7,9)	80.0	5.0	N/A	N/A	N/A	9 (8,9)	94.7	0.0	9 (9,9)	100.0	0.0	N/A	N/A	N/A
		Preventability of the adverse drug event after ICU transfer	8 (6,9)	73.8	7.5	8 (7.75,9)	86.5	2.7	N/A	N/A	N/A	7 (7,8)	84.2	0.0	7 (7,8)	90.0	0.0	N/A	N/A	N/A	9 (8,9)	100.0	0.0	9 (9,9)	100.0	0.0	N/A	N/A	N/A
		Timing of patient adverse drug events on ward after ICU transfer	6 (5,8)	48.1	7.4	6 (5,7)	36.5	4.1	6 (5,7)	35.2	2.8	7 (5,8)	52.6	21.1	7 (5,8)	65.0	15.0	8 (5,9)	73.7	5.3	9 (7,9)	100.0	0.0	9 (7,9)	93.8	0.0	9 (7,9)	94.1	0.0
		Severity of actual harm associated with the adverse drug events occurring on the ward after ICU transfer	8 (6,9)	70.0	6.3	8 (7,9)	81.1	4.1	N/A	N/A	N/A	8 (7,9)	78.9	0.0	8 (7,9)	90.0	0.0	N/A	N/A	N/A	9 (8,9)	100.0	0.0	9 (9,9)	100.0	0.0	N/A	N/A	N/A
		Adverse drug event attributed to ICU or hospital ward care (number documented for patient)	7 (6,9)	68.8	3.8	7.5 (7,9)	79.7	2.7	N/A	N/A	N/A	7 (6,9)	68.4	5.3	7 (6.25,9)	75.0	5.0	N/A	N/A	N/A	8 (7,9)	84.2	0.0	9 (8,9)	100.0	0.0	N/A	N/A	N/A
		Adverse drug events within 30 days of hospital transfer (number documented for patient)	6 (5,8)	42.5	11.3	6 (5,7)	33.8	5.4	6 (5,7)	39.4	4.2	7 (5,7.75)	55.0	5.0	7 (5,7.75)	70.0	0.0	7 (6,8)	68.4	0.0	8 (7,9)	83.3	0.0	8 (8,9)	88.2	0.0	8 (8,9)	94.7	0.0
		Adverse drug events within 90 days of hospital transfer (number documented for patient)	6 (4,7)	39.2	20.3	6 (4.5,7)	28.8	13.7	6 (5,7)	25.7	10.0	5.5 (4,6)	20.0	15.0	6 (4,6.75)	25.0	5.0	6 (5,7)	26.3	0.0	8 (7,9)	78.9	0.0	8 (7,9)	83.3	0.0	8 (7,9)	89.5	0.0
		Adverse drug events identified at post-ICU follow up clinic (number documented for patient)	7 (5,7)	50.6	11.4	7 (5,7)	60.8	8.1	7 (6,7)	64.8	8.5	6.5 (4.25,8)	50.0	5.0	6 (5,7.75)	45.0	0.0	6 (6,7)	47.4	0.0	8 (8,9)	89.5	0.0	8 (8,9)	88.2	0.0	8 (7,9)	94.7	0.0
	B	Clinical frailty scale on ICU admission (older people only, 65+ years)	7 (5,8)	52.6	9.0	7 (5,7)	62.9	2.9	7 (6,7)	72.5	0.0	7 (6,9)	55.6	11.1	7 (6,9)	61.1	5.6	7 (6.75,8.25)	77.8	5.6	8.5 (7,9)	92.9	0.0	8 (7,9)	100.0	0.0	9 (7,9)	100.0	0.0
		Clinical frailty scale on ICU transfer (older people only, 65+ years)	6.5 (4,8)	50.0	12.8	7 (5,7)	58.6	5.7	7 (6,7)	69.6	1.4	6.5 (4,9)	50.0	16.7	7 (6,8.25)	66.7	5.6	7 (6,8.25)	72.2	5.6	8.5 (7,9)	92.9	0.0	9 (7.5,9)	100.0	0.0	9 (8,9)	100.0	0.0
		Clinical frailty scale on hospital discharge (older people only, 65+ years)	6.5 (5,8)	50.0	10.5	7 (5,7)	55.7	5.7	7 (6,7)	69.1	2.9	6 (3.75,9)	44.4	22.2	6 (5,9)	44.4	16.7	7 (5,9)	61.1	16.7	8.5 (7,9)	92.9	0.0	9 (7.5,9)	100.0	0.0	9 (8,9)	100.0	0.0
		Clinical frailty scale within 90 days of hospital discharge	6 (4,7)	45.3	16.0	6 (5,7)	42.6	5.9	6 (5,7)	44.6	3.1	6 (3,7.25)	33.3	27.8	6 (3.75,7.25)	27.8	22.2	6 (5,6.25)	22.2	11.1	7 (6,9)	66.7	0.0	7 (6,8.25)	71.4	0.0	8 (7,9)	93.3	0.0
		Quality of Life measures	7 (5,8)	59.5	2.7	7 (6,8.25)	72.9	0.0	7 (7,8)	80.3	0.0	7 (6,8)	52.6	15.8	6.5 (5.75,8)	50.0	16.7	7 (6,8)	55.6	16.7	8 (7,9)	83.3	5.6	8 (8,9)	88.2	0.0	9 (8,9)	100.0	0.0
	C	Timing of ICU patient transfer to a hospital ward	7 (6,8.5)	66.7	3.7	7 (7,8)	78.4	1.4	N/A	N/A	N/A	8 (6,8)	63.2	10.5	8 (6.25,8)	75.0	5.0	N/A	N/A	N/A	8 (7,9)	84.2	0.0	8 (7,9)	94.4	0.0	N/A	N/A	N/A
		Readmission rate to ICU (within 48 hours)	8 (7,9)	80.2	1.2	8 (8,9)	91.9	1.4	N/A	N/A	N/A	7 (6.25,9)	75.0	0.0	7 (7,9)	90.0	0.0	N/A	N/A	N/A	9 (8,9)	94.4	0.0	9 (8.75,9)	100.0	0.0	N/A	N/A	N/A
		Readmission rate to ICU (within 7 days)	7 (6,8)	64.6	4.9	7 (7,8)	82.4	2.7	7 (7,8)	88.7	1.4	7 (6,8)	60.0	0.0	7 (6,7)	60.0	0.0	7 (7,7)	78.9	0.0	7 (7,9)	82.4	0.0	7 (7,8.25)	100.0	0.0	7 (7,9)	100.0	0.0
		Readmission rate (unplanned) to hospital (within 30 days)	7 (5,8)	56.8	6.2	7 (6,8)	68.5	1.4	7 (7,8)	80.0	1.4	6 (6,9)	47.4	5.3	6.5 (6,7)	50.0	0.0	7 (6,8)	52.6	0.0	7.5 (5.75,9)	66.7	0.0	7 (7,8)	82.4	0.0	7 (7,9)	94.7	0.0
		Readmission rate (unplanned) to hospital (within 90 day)	5 (4,7)	39.2	20.3	6 (5,7)	38.9	9.7	6 (5,7)	40.0	5.7	6 (4,7)	26.3	10.5	6 (6,6)	15.8	5.3	6 (5,6)	21.1	5.3	6.5 (5,8.25)	50.0	0.0	7 (5.5,7.5)	70.6	0.0	7 (6,7)	68.4	0.0
		Resuscitation team call on ward after ICU transfer to the hospital ward	7 (5,9)	61.5	7.7	8 (6,9)	74.6	5.6	N/A	N/A	N/A	8 (6.25,9)	75.0	0.0	8.5 (6.5,9)	75.0	0.0	N/A	N/A	N/A	9 (8,9)	94.1	0.0	9 (9,9)	100.0	0.0	N/A	N/A	N/A
	D	Length of patient hospital stay post-ICU transfer	7 (5,8)	54.3	4.9	7 (6,7.25)	63.5	0.0	7 (6,8)	69.0	0.0	7 (5.25,8)	55.0	10.0	7 (6,7.75)	60.0	5.0	7 (6,8)	73.7	5.3	8 (7,9)	80.0	0.0	8 (7,8.25)	88.9	0.0	8 (7,9)	94.7	0.0
		Length of patient hospital stay	7 (5,8)	53.1	4.9	7 (6,8)	63.5	1.4	7 (6,8)	69.0	0.0	7 (6,8)	55.0	15.0	6.5 (6,8)	50.0	5.0	7 (6,8)	57.9	5.3	8 (7,9)	84.2	0.0	8 (7,9)	88.2	0.0	8 (7,9)	94.7	0.0
		Mortality rate (within 7 days of patient ICU transfer to a hospital ward)	7 (5,8)	59.0	3.6	7 (6,9)	71.6	0.0	N/A	N/A	N/A	8 (7,9)	85.0	0.0	8 (7,9)	90.0	0.0	N/A	N/A	N/A	9 (7,9)	94.4	0.0	9 (8,9)	100.0	0.0	N/A	N/A	N/A
		Mortality rate (within 30 days of patient ICU transfer to a hospital ward)	7 (5,8)	53.7	3.7	7 (6,8)	64.9	0.0	7 (7,8)	81.7	0.0	7 (6,8.75)	65.0	0.0	7 (6.25,8.75)	75.0	0.0	7 (6,9)	73.7	0.0	8 (7,9)	83.3	0.0	8 (7,9)	82.4	0.0	8 (7,9)	94.4	0.0
		Mortality rate (within 90 days of patient ICU transfer to a hospital ward)	6 (5,8)	41.3	11.3	6 (5,7)	37.8	4.1	6 (5,7)	38.0	0.0	6 (5,7)	45.0	10.0	6 (5.25,7)	45.0	5.0	6 (6,7)	47.4	5.3	7 (5,8)	66.7	0.0	7 (5.5,8)	75.0	0.0	7 (6.75,8)	77.8	0.0
		Mortality rate (within 30 days of hospital discharge)	6 (5,8)	46.3	11.0	6 (5,7)	47.3	4.1	7 (6,7)	57.7	2.8	7 (4.25,8.75)	55.0	10.0	7 (5,8.5)	60.0	0.0	7 (5,9)	63.2	0.0	8 (6.75,9)	77.8	0.0	7 (7,8)	81.3	0.0	7 (7,8)	94.4	0.0
		Mortality rate (within 90 days of hospital discharge)	5.5 (5,8)	40.0	16.3	6 (5,7)	33.8	9.5	6 (5,7)	38.0	1.4	6 (4.25,7)	40.0	20.0	6.5 (5,7)	50.0	15.0	7 (5,8)	57.9	10.5	7 (5,8)	66.7	0.0	7 (6,7)	68.8	0.0	7 (6,7)	66.7	0.0
